# Expert Opinion on Allogeneic Bone Marrow-Derived Mesenchymal Stem Cell (BMMSC) Therapy for Knee Osteoarthritis: Clinical Insights, Efficacy, and Safety

**DOI:** 10.7759/cureus.107926

**Published:** 2026-04-28

**Authors:** Sanjay B Londhe, Vijay Goni, Ravi Shah, Ravi Sauhta, Pardeep Bageja, Natesh Kolusu, Vijal Modi, Bishal Bhagat, Rajashekar Danda, Gaurav Bhandari, Girish Bhalerao, Krishna Shriram Dhanasekaran, Prashant Palkar, Sucheta Mehta

**Affiliations:** 1 Department of Orthopedics, Criticare Asia Hospital, Mumbai, IND; 2 Department of Orthopedics and Traumatology, Postgraduate Institute of Medical Education and Research, Chandigarh, IND; 3 Department of Orthopedics, Sujay Hospital, Mumbai, IND; 4 Department of Orthopedics and Joint Replacement, Artemis Hospital Gurgaon, Gurgaon, IND; 5 Department of Orthopedics, Sir Ganga Ram Hospital, New Delhi, IND; 6 Department of Orthopedics and Trauma, Virinchi Hospitals, Hyderabad, IND; 7 Department of Orthopedics, Dr. Vijal Modi (DVM) Hospital, Ahmedabad, Ahmedabad, IND; 8 Department of Orthopedic Surgery, Manipal Hospitals, Kolkata, Kolkata, IND; 9 Department of Orthopedics, PRK Super Specialty Hospitals, Hyderabad, Hyderabad, IND; 10 Department of Orthopedics and Joint Replacement, Dharamshila Narayana Superspeciality Hospital, New Delhi, IND; 11 Department of Orthopedics and Joint Replacement, Gleneagles Hospitals, Mumbai, IND; 12 Department of Medical Affairs, Cipla Ltd., Mumbai, IND

**Keywords:** cartilage preservation, clinical consensus, knee osteoarthritis, orthobiologics, regenerative medicine

## Abstract

Background: Knee osteoarthritis (KOA) remains a major cause of disability. Many patients with moderate disease (Kellgren-Lawrence (KL) grade 2-3) continue to experience disease progression, despite receiving guideline-based conservative treatments that primarily offer symptomatic relief. Allogeneic bone marrow-derived mesenchymal stem cells (BMMSCs) have gained attention for their potential anti-inflammatory, immunomodulatory, and chondroprotective effects. This consensus exercise aimed to capture expert perspectives on diagnostic practices, clinical utility, efficacy, safety, and real-world integration of allogeneic BMMSC therapy.

Methods: A structured expert panel comprising experienced orthopedic surgeons reviewed preclinical, clinical, and regulatory evidence supporting allogeneic BMMSCs for KOA. Real-time online polling collected quantitative data on diagnostic approaches, perceived efficacy, safety experience, cartilage-regeneration assessment, and broader orthopedic indications.

Results: Most experts (81.8%) relied on physical examination and X-ray (KL grading) for diagnosis, while MRI was used selectively. A majority (72.7%) considered allogeneic BMMSCs effective for KL grade 2-3 KOA. Routine MRI monitoring for cartilage regeneration was uncommon; only 9.0% reported performing post-procedural MRI for visualizing structural improvements. No severe allergic or anaphylactic reactions were reported, and adverse events were described as mild and transient. Regulatory approval and GMP/GLP-certified manufacturing were viewed as key contributors to both clinician and patient confidence and insurance coverage. Experts supported the use of allogeneic MSCs for cartilage defects and, to a lesser extent, avascular necrosis and tendon or ligament injuries. Identified gaps included long-term durability and standardized rehabilitation protocols.

Conclusions: The expert panel found that allogeneic BMMSCs appear to be a safe and effective treatment for grade 2-3 KOA. Additional real-world data and targeted guidelines are needed to better define the role of allogeneic BMMSCs in routine orthopedic practice.

## Introduction

Osteoarthritis (OA) is a highly prevalent degenerative joint disease worldwide. Its burden increases with age, with about 73% of affected individuals being older than 55 years, and it is more common in women, who account for approximately 60% of cases [1]. The knee is among the most affected joints, and its degeneration often increases with advancing age. However, age alone does not fully account for the rising knee OA burden; the global increase in obesity and metabolic disorders has emerged as a major contributing factor that accelerates joint degeneration beyond normal wear-and-tear [2]. Clinically, knee OA presents with characteristic symptoms and signs, including pain, stiffness, joint tenderness, bony enlargement, crepitus, reduced range of motion, and, in advanced cases, muscle wasting around the joint. These clinical features, along with supporting radiographic findings of joint degeneration, are used to diagnose knee OA [3].

Current management strategies for knee OA typically follow a stepwise approach, including non-surgical measures such as patient education, exercise, weight management, and symptomatic relief with non-steroidal anti-inflammatory drugs (NSAIDs); when needed, intra-articular injections (viscosupplementations, hyaluronic acid, and corticosteroids) and surgical interventions (osteotomy or arthroplasty) are reserved for advanced disease [4,5]. While these conventional therapies can reduce pain and improve function, they do not stop the disease progression, i.e., progressive cartilage damage. As a result, many patients, particularly those with grade 2-3 knee OA, remain symptomatic, and in those requiring long-term pharmacologic therapy, the risk of adverse effects increases [6].

In recent years, regenerative approaches have gained interest for their potential to not only relieve symptoms but also to preserve or partially restore joint structure. Among these, mesenchymal stem cell (MSC)-based therapy stands out. Preclinical and clinical studies have suggested that MSC implantation, especially allogeneic MSCs, may offer pain relief, functional improvement, immunomodulation, and chondroprotection and chondropreservation through possible regeneration [7]. Clinical trials have reported sustained improvements in pain and function up to 24 months. This preclinical evaluation confirmed the safety and efficacy of haMSCs, with intra-articular injection leading to improvements in pain, function, and knee cartilage volume. The 5 × 10⁷ haMSC dose demonstrated the most pronounced therapeutic benefit [8].

Given this background, the present expert consensus meeting was convened to evaluate the safety and effectiveness of intra-articular allogeneic bone marrow-derived MSC (BMMSC) therapy in patients with knee OA who remain symptomatic despite standard-of-care conservative treatment. Specifically, we aimed to assess changes in pain (using VAS (Visual Analog Scale)), functional outcomes (e.g., WOMAC (Western Ontario and McMaster Universities Arthritis Index) or KOOS (Knee injury and Osteoarthritis Outcome Score)), maintenance of cartilage quality, and occurrence of adverse events, thereby contributing real-world data to inform whether allogeneic BMMSC therapy might serve as a viable joint-preserving option in knee OA.

## Materials and methods

Study design

A structured expert panel discussion was organized to review and validate the scientific rationale, manufacturing process, preclinical data, and clinical evidence on allogeneic BMMSCs for the management of knee OA. The meeting aimed to gather clinical insights, standardize treatment protocols, and support evidence generation and consensus building.

Panel composition

Panel Selection Criteria

Orthopedic surgeons with at least 10 years of clinical experience from tertiary care hospitals and specialized orthopedic centers from diverse geographic regions and practice settings across India were included. A total of 11 orthopedic surgeons meeting these criteria participated in the expert opinion exercise.

Panelists/Participants Involved

Author identities and institutional affiliations are transparently listed in the manuscript to ensure accountability and disclosure.

Consensus Methodology

The expert opinion paper is of a non-Delphi nature of the consensus process to avoid methodological ambiguity and clarifies that the intent was to reflect real-world clinical perspectives, not to generate formal clinical guidelines. The exercise employed a structured discussion framework, followed by real-time, anonymized online polling, during the physical scientific advisory board meeting with the 11 panelists. The expert opinion exercise was conducted as a single structured in-person scientific meeting for 3.30 hours, during which predefined clinical evidence and discussion domains were reviewed.

Questionnaire Design

Polling questions were developed by the organizing scientific team based on key clinical decision points in knee OA management, existing literature on MSC-based therapies, and regulatory considerations specific to the Indian context. Questions included multiple choices focused on practice patterns and expert perceptions, rather than forcing binary judgments. Polling questions were finalized in advance and administered in a single round without iteration or re-voting.

Data Analysis

Real-time polling was performed using an anonymized electronic platform to collect independent responses from panel members. Responses were analyzed descriptively, without predefined consensus thresholds, reflecting the intent to capture expert perspectives and real-world practice patterns rather than to generate formal clinical guidelines.

Discussion Framework

The discussion was structured around the following domains: patient selection criteria, pre-medication and administration protocol, efficacy and safety review, radiological outcomes, post-injection rehabilitation, polling and data collection, and need for awareness initiatives among doctors and patients. An interactive online polling system was used during the session to collect real-time opinions on diagnostic practices for knee OA, perceived clinical efficacy and safety of allogeneic BMMSCs, observations on radiographic efficacy: cartilage regeneration, experience with adverse events, increased confidence due to Indian regulatory (Drugs Controller General of India (DCGI)) approval, potential for other orthopedic applications, and preference ranking among stem cell therapies. The responses were documented for qualitative analysis and will contribute to the development of the Expert Opinion paper.

Ethical considerations

The meeting followed ethical principles for expert consultations, ensuring transparency, voluntary participation, and non-promotional intent. Individual responses to the poll were anonymized for analysis.

Planned outcomes

The primary objective was to capture the clinical perspectives of experienced orthopedic surgeons on the role of BMMSC therapy in the management of grade 2-3 knee OA in real-world practice.

The secondary objectives were to understand (1) expert perspectives on patient selection criteria and diagnostic approaches used prior to allogeneic BMMSC therapy; (2) perceived clinical efficacy and safety based on real-world experience; (3) current practices related to structural assessment and cartilage regeneration monitoring; (4) the impact of Indian regulatory (DCGI) approval on clinician confidence and treatment standardization; and (5) potential orthopedic indications beyond knee OA and highlight existing evidence gaps.

## Results

Expert insights and poll analysis

A total of 11 doctors (Sanjay B. Londhe, Vijay Goni, Ravi Shah, Ravi Sauhta, Pardeep Bageja, Natesh Kolusu, Vijal Modi, Bishal Bhagat, Rajashekar Danda, Gaurav Bhandari, and Girish Bhalerao) opined in the consensus.

Diagnosis of OA

Panel Recommendation

Almost nine (81.8%) of experts diagnose knee OA based on clinical symptoms and X-ray (KL grading), while four (36.4%) rely on knee MRI.

OA diagnosis remains fundamentally clinical and radiographic, supported by strong evidence and major international guidelines. The 1986 American College of Rheumatology (ACR) criteria, which integrate age, symptoms, physical examination, and radiographic/laboratory findings, continue to be widely referenced in practice, although newer guidelines such as the 2019 ACR/Arthritis Foundation Guideline shift focus toward broader evidence-based management rather than strict radiographic thresholds [9]. European recommendations from the EULAR Task Force emphasize that OA can often be confidently diagnosed clinically using three key symptoms (persistent pain, brief morning stiffness, and reduced physical function) and three characteristic signs (crepitus, restricted movement, and bony enlargement) [10]. Despite advances in imaging, plain X-ray remains the most practical tool because it is inexpensive, accessible, and standardized [11]. The Kellgren-Lawrence (KL) grading system (grades 1-4), based on osteophytes, joint-space narrowing, and subchondral sclerosis, is globally endorsed by the ACR, Osteoarthritis Research Society International (OARSI), and European Alliance of Associations for Rheumatology (EULAR) and correlates well with disease severity, symptom burden, and treatment decisions, distinguishing mild/moderate OA from severe, treatment-refractory disease [9,11-13]. While MRI provides superior visualization of cartilage, synovium, and bone marrow lesions, its routine use is limited by cost, availability, and its lack of necessity in typical presentations; thus, MRI is mainly reserved for atypical, complex, or early OA cases [14,15]. More invasive tools, such as arthroscopy or histology, offer direct visualization but are impractical for diagnosis. Overall, the integration of clinical symptoms, physical examination, and X-ray KL grading remains the practical, high-value, evidence-supported approach for diagnosing knee OA across clinical settings [16,17].

Efficacy of allogeneic BMMSC

Panel Recommendation

Approximately eight (72.7%) of experts agreed that stem cell therapy is effective in patients with grades 2 and 3 knee OA, while three (27.2%) were uncertain.

Patient selection criteria in clinical practice: The doctors agreed that patients with KL grade 2 or 3 knee OA meeting the following criteria are suitable for treatment with allogeneic BMMSCs: (a) body mass index (BMI) < 30 kg/m² or body weight < 85 kg; (b) absence of inflammatory arthritis and active infections; (c) varus or valgus deformity < 5°; (d) no significant mechanical deformity; and (e) glycated hemoglobin (HbA1c) < 8%.

Preclinical and Phase II and III clinical studies of Stempeucel® have demonstrated its capacity to differentiate into chondrocytes, showing downregulation of Sox9 and upregulation of Col2A, with production of sulfated glycosaminoglycans (sGAG) and other cartilage matrix components in vitro, suggesting a potential for cartilage repair or preservation rather than mere symptomatic relief [18].

In a randomized, double-blind, multicenter, placebo-controlled Phase III trial of allogeneic BMMSC therapy (Stempeucel®) in patients with knee OA, MSC treatment led to significant clinical improvements; patients receiving Stempeucel® showed marked reductions in pain and functional impairment as demonstrated by significant decreases in WOMAC total score at six months and even greater reductions at 12 and 24 months (mean change of −23.6% at six months and −45.6% at 12 months versus placebo; P < 0.001). MRI T2-mapping revealed no worsening of deep cartilage in the MSC group over 24 months, while the placebo group experienced progressive cartilage degeneration (P < 0.001), and cartilage volume remained stable in the allogeneic MSC-treated knees [19].

Safety was acceptable; only a small number of drug-related adverse events, mostly mild injection-site swelling or pain, were reported, which resolved within days, highlighting a favorable tolerability profile and supporting the anti-inflammatory and functional benefits of MSC therapy in OA [20]. Additionally, a recent 2025 meta-analysis of randomized controlled trials of intra-articular MSC therapy (n=502 patients) confirmed significant improvements in pain (VAS) and functional scores (WOMAC, KOOS) at six and 12 months compared to control, with no increase in adverse events, reinforcing the growing evidence that MSC therapy is a viable, safe, and efficacious option for knee OA management [21].

Cartilage regeneration with allogeneic BMMSCs

Panel Recommendation

The majority of panelists (10, 90.9%) reported that they had not performed repeat MRI follow-ups to assess cartilage regeneration with allogeneic BMMSCs, as patients tend to avoid follow-up MRIs once they feel asymptomatic and well, due to their cost. While one (9.0%) performed MRI and observed cartilage regeneration.

MSC-based therapies have shown the ability to generate cartilage-like (bionic) tissues that replicate the structural and functional characteristics of native articular cartilage, largely due to their strong chondrogenic potential [21]. MSCs delivered intra-articularly can prevent or delay cartilage degeneration, reduce pain, and improve joint function in both osteoarthritis and inflammatory arthropathies, as demonstrated in preclinical and clinical studies [22].

Thrombospondin-2 (TSP-2) is an extracellular matrix protein and prochondrogenic factor that plays a major role in determining the chondrogenic differentiation potential of MSCs. Allogeneic BMMSCs are analyzed for their ability to secrete TSP-2 [19].

In the Phase 3 Extension Clinical trial, at one- and two-year follow-up, cartilage quality was maintained in the deep cartilage of the medial compartment (the most commonly affected in osteoarthritis), with a mean T2 relaxation time of <38 ms in the allogeneic BMMSC arm, whereas in the placebo arm, it increased to 47 ms, indicating the continuous degeneration of cartilage. Also, cartilage volume increased in the allogeneic BMMSC arm at two years, whereas it decreased in the placebo arm [19]. In a Spanish study, feasibility and safety were established, with evidence of clinical efficacy. MSC-treated patients achieved superior functional improvement compared with active controls receiving hyaluronic acid and showed significant improvements in cartilage quality and volume, as assessed by T2 relaxation mapping [23].

**Figure 1:**
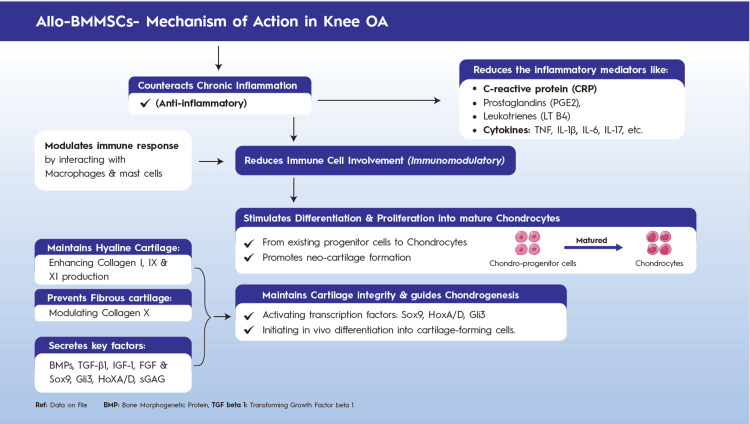
Mechanism of action of allogeneic BMMSCs in knee OA Allo-BMMSCs: Allogeneic Bone Marrow-derived Mesenchymal Stem Cells; BMPs: Bone Morphogenetic Proteins; CRP: C-Reactive Protein; FGF: Fibroblast Growth Factor; Gli3: Glioma-Associated Oncogene Homolog 3; HoxA/D: Homeobox A/D Genes; IGF-1: Insulin-Like Growth Factor 1; IL-1β: Interleukin-1 Beta; IL-6: Interleukin-6; IL-17: Interleukin-17; LT B4: Leukotriene B4; OA: Osteoarthritis; PGE2: Prostaglandin E2; sGAG: Sulfated Glycosaminoglycans; Sox9: SRY-Box Transcription Factor 9; TGF-β1: Transforming Growth Factor Beta 1; TNF: Tumor Necrosis Factor. Image created by the authors using Adobe Illustrator.

A major component of their therapeutic efficacy stems from paracrine mechanisms, through which MSC-secreted factors inhibit chondrocyte apoptosis, reduce inflammation, and support ongoing cartilage maintenance [21]. Chondrogenic growth factors further modulate MSC differentiation, proliferation, and metabolic activity, enhancing the formation of new cartilage matrix and improving cartilage stability. When used as cell-rich MSC matrices through surgical delivery, MSCs promote the regeneration of both hyaline cartilage and fibrocartilage, rebuilding damaged articular surfaces more effectively than traditional microfracture techniques. BMMSCs secrete potent bioactive factors that protect existing cartilage from enzymatic degradation and activate endogenous progenitor cells to stimulate intrinsic cartilage repair [24]. This mechanism of action is described in Figure 1.

Clinically, these regenerative and chondroprotective mechanisms have translated into measurable structural preservation: in a Phase III randomized trial, Gupta et al. reported that deep cartilage in the medial femorotibial compartment showed no worsening at 12 and 24 months following allogeneic BMMSC treatment, whereas the placebo group exhibited progressive, statistically significant cartilage deterioration (P < .001) [25].

Safety of allogeneic BMMSCs

Panel Recommendation

All the experts reported that they had not witnessed any severe allergic or anaphylactic reactions after using allogeneic BMMSCs, while only one (9.0%) had observed minor injection-site reactions like swelling and redness.

**Figure 2:**
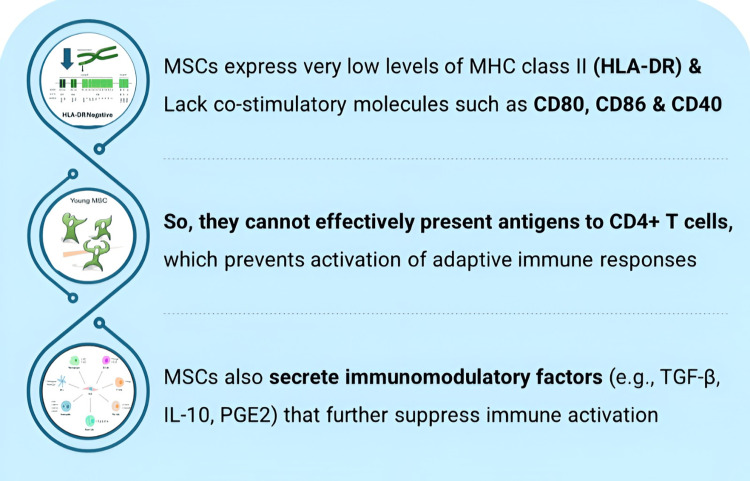
Allogeneic BMMSCs are immune privileged HLA-DR: Human Leukocyte Antigen – DR Isotype; IL-10: Interleukin 10; MHC: Major Histocompatibility Complex; MSCs: Mesenchymal Stem Cells; PGE2: Prostaglandin E2; TGF-β: Transforming Growth Factor Beta. Image created by the authors using Adobe Illustrator.

Reason: MSCs lack the expression of hematopoietic markers CD45, CD34, CD14, or CD11b, CD79a or CD19, and MHC Class II: HLA-DR. MSCs modulate immunosuppression and interact with many immune cells, including B cells, T cells, dendritic cells (DCs), natural killer (NK) cells, and macrophages. Mechanisms of interaction rely on either the secretion of immunomodulatory factors like prostaglandin E2, indoleamine 2,3-dioxygenase (IDO), nitric oxide (NO), and transforming growth factor-β (TGF- β), or cell-cell interaction, thus making them ideal cell types for the treatment of OA (Figure 2) [26].

A PRISMA-guided review of clinical studies from PubMed, Scopus, and EMBASE consistently shows that allogeneic MSC implantation for knee osteoarthritis demonstrates an excellent safety profile, with adverse events largely limited to mild, transient local reactions and no long-term effects [20].

Across both Phase II and Phase III randomized, double-blind trials of intra-articular Stempeucel® (allogeneic BMMSCs), no serious treatment-related adverse events were reported, and the most frequent reactions, brief injection-site swelling or discomfort, typically resolved spontaneously within two to three days, without the need for any medical intervention [26]. The Phase III trial specifically documented only five possibly or probably product-related adverse events, all mild local effects, with no systemic toxicity, immune reactions, infections, or discontinuations linked to the investigational therapy [20]. Consistent findings were reported in international studies; a Spanish clinical trial of allogeneic BMMSCs by the University of Valladolid showed good tolerability, with only self-resolving local discomfort and no immunogenic or serious adverse events following intra-articular administration [23], while Korean trials evaluating allogeneic BMMSCs for knee OA similarly reported excellent safety and no severe adverse events, further supporting the low immunogenicity and high tolerability of allogeneic BMMSC therapy [26]. Additionally, early-phase clinical investigations of intra-articular adipose-derived MSCs (AD-MSCs) demonstrate a comparable safety profile, with early evidence of safety and potential cartilage regeneration, although larger randomized controlled studies are needed for confirmation [27]. Collectively, current evidence strongly supports the favorable safety and tolerability of allogeneic BMMSC-based therapies for knee OA [28].

Indian regulatory approval for allogeneic BMMSC for knee OA

Panel Recommendation

All experts agreed that the approval of allogeneic BMMSC for knee OA by the Indian regulatory authority, the DCGI, increased their confidence in its safety and efficacy for using it as a treatment option. They also felt that it was standardized therapy due to regulatory approval, with the manufacturing process approved by the Central Drugs Standard Control Organisation (CDSCO) following Good Manufacturing Practice (GMP) in a GLP-certified laboratory.

Furthermore, doctors opined that regulatory approval has made this procedure easy for Indian patients to claim under health insurance.

In September 2022, India became the first country to authorize the commercial use of an allogeneic BMMSC product for knee OA (grade 2 and 2), following regulatory approval by the DCGI, as reported by national biomedical news outlets and official statements from Stempeutics Research Pvt. Ltd. [29].

Multiple systematic reviews examining the efficacy and safety of MSC-based therapy for knee OA have consistently found no major safety concerns, with adverse events largely limited to mild, transient injection-site discomfort and no reports of significant immunogenicity or long-term complications [30]. Reflecting this trend, expert commentary from leading Indian orthopedic clinicians has highlighted that allogeneic stem-cell therapy represents a promising therapeutic option, especially in the early stages of knee OA or in patients who have exhausted standard non-surgical measures, emphasizing its emerging role in joint preservation strategies in Indian orthopedic practice [31].

Allogeneic MSCs and their potential to treat other orthopedic indications

Panel Recommendation

Ten (90.9%) experts considered allogeneic MSCs for cartilage defects, while five (45.4%) also considered them for chondromalacia patellae, avascular necrosis, tendon injuries, and ligament injuries.

Global evidence on allogeneic MSC therapy shows encouraging outcomes across multiple musculoskeletal indications. In knee articular cartilage defects, a randomized controlled trial demonstrated that umbilical cord-derived MSCs with hyaluronate (UCB-MSC-HA) produced superior cartilage repair, pain reduction, and functional improvement over microfracture, with benefits sustained up to five years [29].

In hip avascular necrosis, outcomes depend on disease stage, lesion volume, and MSC dose; studies show that injecting higher-than-optimal cell doses does not confer additional benefit, emphasizing the importance of tailored cell concentrations. For tendon and ligament pathologies, allogeneic MSCs have demonstrated the ability to modulate inflammation, reduce fibrosis, and enhance collagen organization, improving overall healing quality in both preclinical and early clinical studies [30-33].

In ACL reconstruction, a prospective study evaluating intra-articular and intra-tunnel delivery of allogeneic MSCs reported that the treatment was safe and showed promising improvements in symptoms, graft maturation, and tunnel healing over 24 months, supporting their potential as an adjunct to surgical repair [30-33].

First preference among stem cell therapies for knee OA

Panel Recommendation

All doctors agreed that allogeneic BMMSCs were their first choice for stem cell therapy. They opined that the available safety and efficacy clinical data, regulatory approval, standardization of dosing, and off-the-shelf availability are the reasons.

MSCs have emerged as a practical and effective alternative to autologous MSCs for chronic knee OA, offering off-the-shelf availability and consistent cell quality [26].

Autologous and allogeneic MSCs: Both autologous and allogeneic MSCs show promising results in OA treatment. Whereas allogeneic BMMSCs derived from three healthy donors are pooled and expanded, they exhibit enhanced growth kinetics and a greater propensity for chondrogenic differentiation. They appear to be a viable option that can be readily used as an ‘off-the-shelf’ product without concerns about local site morbidity, cell variability issues due to factors such as aging and comorbidities, or the time required for processing autologous MSCs [26].

In a Phase 3 randomized, double-blind, multicenter, placebo-controlled trial conducted by Gupta et al., T2-mapping revealed that deep cartilage in the medial femorotibial compartment remained stable in the allogeneic BMMSC-treated group over 12 and 24 months, while the placebo group showed a progressive and significant deterioration (P < .001) [18].

The summary of recommendations is demonstrated in Table 1.

**Table 1:** Summary of recommendations BMMSC/BMMSCs: Bone Marrow-derived Mesenchymal Stem Cell(s); DCGI: Drugs Controller General of India; HLA-DR2: Human Leukocyte Antigen DR2; KL: Kellgren–Lawrence (grading system); MRI: Magnetic Resonance Imaging; MSC/MSCs: Mesenchymal Stem Cell(s); OA: Osteoarthritis; X-ray: X-radiography

Domain	Expert Panel Consensus
Diagnosis of Knee OA	Knee osteoarthritis is primarily diagnosed using clinical evaluation supported by X-ray with Kellgren–Lawrence grading; MRI is used selectively.
Patient Selection	Allogeneic BMMSC therapy is considered appropriate mainly for patients with symptomatic KL grade 2–3 knee OA who are unresponsive to conservative treatment.
Efficacy	Allogeneic BMMSCs provide meaningful improvements in pain and functional and radiological outcomes in moderate knee osteoarthritis.
Cartilage Regeneration Assessment	Routine MRI follow-up to assess cartilage regeneration is uncommon in clinical practice. But it can be adopted in practice, which will help generate real-world data.
Mechanism of Action	Therapeutic effects are attributed predominantly to paracrine, anti-inflammatory, immunomodulatory, and chondroprotective mechanisms
Safety	No severe allergic or anaphylactic reactions have been observed; reported adverse events are mild, such as injection site reactions
Immunogenicity	Allogeneic BMMSCs demonstrate low immunogenicity in clinical use due to a lack of HLA DR2
Indian Regulatory Approval	The DCGI approval has increased clinician confidence and contributed to treatment standardization.
Other Orthopedic Indications	Allogeneic BMMSCs are considered for cartilage defects and, to a lesser extent, for avascular necrosis and tendon or ligament injuries.
Preferred Stem Cell Source	Allogeneic bone marrow-derived MSCs are the preferred stem cell therapy among the experts
Evidence Gaps	Long-term outcomes (>2 years), active recommendations by all the Guidelines, need for repeat injections, and standardized rehabilitation protocols are needed

## Discussion

The consensus findings, when viewed through the lens of guidelines and clinical evidence, suggest several practical implications for the orthopedic community. MSC therapy appears most appropriate for symptomatic KL grade 2-3 knee OA patients who have not responded to conservative care and who seek joint-preserving options before considering arthroplasty. Diagnostic workflows should continue to emphasize clinical evaluation and X-ray KL grading, reserving advanced imaging for selected cases. The availability of a GMP-certified, regulatory-approved (DCGI) allogeneic MSC product provides a foundation for standardized treatment protocols and insurance integration. But clinicians should also prioritize structured post-procedural rehabilitation and consistent outcome tracking to monitor and maximize therapeutic benefits. Establishing long-term registries, harmonizing dosing protocols, and encouraging multidisciplinary collaboration will be essential steps to strengthen the evidence base and guide the safe, effective, and equitable use of MSC therapies. By advancing clinician education and responsible practice standards, the orthopedic community can help ensure that MSC treatments fulfill their potential without exceeding current evidence.

How do the panel’s responses align with guidelines and professional recommendations?

The panel’s diagnostic preference for combining clinical assessment with plain radiography closely mirrors guidelines and professional recommendations, which emphasize that knee OA remains fundamentally a clinical-radiographic diagnosis. International guidance from ACR and OARSI similarly prioritizes symptoms, physical examination, and X-ray findings over advanced imaging in routine cases, highlighting X-ray KL grading as a practical, evidence-supported approach [9,16,34]. The panel’s endorsement of allogeneic BMMSC therapy for KL grade 2-3 knee OA. It also aligns with emerging professional consensus statements, such as the ESSKA ORBIT Consensus recommendations for cell-based therapies in knee OA, which consider MSC therapy appropriate in carefully selected patients (grade 1-3 knee OA) while acknowledging that it is not yet standard care [35]. Furthermore, the panel’s limited use of repeat MRI to assess cartilage regeneration aligns with the pragmatic stance outlined in guidelines, which reserve MRI for atypical or complex cases rather than for routine monitoring. The increased clinician confidence following the Indian Regulatory (DCGI) approval is also consistent with broader observations that regulatory authorization enhances clinical adoption and adaptability by establishing standardized manufacturing pathways and reassuring both clinicians and patients on the product quality.

Contribution of the panel’s findings to current orthopedic practice

Together, the panel’s findings reflect a shift toward more structured, evidence-based decision-making in orthopedic practice. The consensus also suggests that MSC therapies are gradually being integrated into the continuum of care, particularly for patients who remain symptomatic despite guideline-based conservative treatments. However, the panel’s limited reliance on structural imaging, such as MRI, suggests that much of real-world practice still focuses on symptom improvement rather than objective tissue-level outcomes. Overall, the survey results show that Indian orthopedicians are beginning to adopt MSC therapies within a framework informed by evidence, regulation, and practical considerations. There is also a need for orthopedicians to generate evidence in real-world scenarios, i.e., through integration into their clinical practice.

Strengths of the study

A key strength of this consensus exercise is its alignment with current clinical guidelines and available evidence, particularly regarding the use of clinical assessment and radiographic evaluation for diagnosing knee osteoarthritis. The panel’s views on the role of allogeneic BMMSC therapy were generally cautious, mindful, and grounded in clinical evidence and real-world experience, reflecting practical considerations that clinicians frequently encounter. The discussion also acknowledges the value of regulatory approval, dosing standardization, manufacturing consistency, and appropriate clinical use. The emphasis on safety and outpatient feasibility mirrors observations from their clinical practice and published clinical studies. Collectively, these elements contribute to a balanced, evidence-aware perspective on the integration of allogeneic BMMSC therapy into the orthopedic practice.

Limitations

Despite its strengths, the consensus also has several limitations that should be acknowledged. First, significant evidence gaps remain, particularly regarding the long-term durability of MSC therapy, rare adverse events, the need for repeat injection strategies, and standardized rehabilitation protocols. The discussion centered on standardized, DCGI-approved allogeneic BMMSC therapy; a degree of preference bias toward allogeneic approaches cannot be excluded. Practical challenges, including higher cost and reliance on cold-chain logistics, may restrict widespread adoption. However, recent inclusion in the insurance claims process can subtly overcome the cost concern. These limitations underscore the need for long-term registries, comparative studies, and health economic evaluations to improve the responsible and equitable use of allogeneic MSC therapies.

## Conclusions

This expert opinion exercise suggests that allogeneic BMMSC therapy may have potential clinical utility as an effective joint-preserving option for carefully selected patients with grade 2-3 knee OA who remain symptomatic despite conservative treatment. This also reflects expert interpretation of existing evidence and their real-world experience, rather than direct efficacy data generated by this consensus exercise. While current clinical experience supports an acceptable safety profile within a standardized, regulatory-approved framework, important evidence gaps remain, particularly regarding long-term outcomes (beyond two years), any need for repeat injection, and standardized rehabilitation protocols. Further real-world data and focused clinical guidelines are needed to better define the role of allogeneic BMMSCs in routine orthopedic practice.
